# Diagnosis of Bilateral Tonsil Cancers via Staging PET/CT: Case Report and Review

**DOI:** 10.1155/2011/928240

**Published:** 2011-07-14

**Authors:** Edward M. Mannina, Sunanda M. Pejavar, Christine M. Glastonbury, Annemieke van Zante, Steven J. Wang, Sue S. Yom

**Affiliations:** ^1^Department of Radiation Oncology, University of California San Francisco, 1600 Divisadero Street, P.O. Box 1708, Suite H-1031, San Francisco, CA 94143, USA; ^2^Department of Radiology & Biomedical Imaging, University of California San Francisco, 505 Parnassus Avenue, P.O. Box 0628, Room L-358, San Francisco, CA 94143, USA; ^3^Department of Anatomic Pathology, University of California San Francisco, 1600 Divisadero Street, P.O. Box 1785, Room B231, San Francisco, CA 94143, USA; ^4^Department of Otolaryngology-Head and Neck Surgery, University of California San Francisco, 2233 Post Street, 3rd Floor, P.O. Box 1225, San Francisco, CA 94115, USA

## Abstract

Diagnostic workup of metastatic head and neck squamous cell carcinoma of unknown primary site has traditionally included CT and/or MRI imaging and endoscopic biopsies. Routine bilateral tonsillectomy is highly controversial and the role of PET/CT is evolving, both for identification of potential primary sites and the detection of distant metastases. We report a case of cervical nodal metastasis of squamous cell carcinoma from an unknown primary site, in which dual-modality PET/CT led to the unexpected diagnosis of synchronous bilateral tonsillar cancers. In addition, PET/CT correctly distinguished pulmonary sarcoidosis from metastatic disease in this patient.

## 1. Introduction

The standard workup for a head and neck squamous cell carcinoma of unknown primary site (CUPS) includes physical exam, chest imaging, CT or MRI of the head and neck region, and panendoscopy with biopsies of potential primary sites. Given that a high proportion of occult tumors are located in the palatine tonsils, diagnostic unilateral tonsillectomy is frequently recommended. Bilateral tonsillectomy has been proposed but remains controversial. This case illustrates that skilled interpretation of PET/CT, incorporating a diagnostic-quality anatomic imaging component, can correctly identify clinically inapparent synchronous tonsil cancers and assist in the evaluation for distant disease.

## 2. Case Report

A 57-year-old man presented with a flu-like syndrome and right neck swelling. Fine-needle aspiration of the right neck mass revealed squamous cell carcinoma, and a CT scan of the head and neck revealed an enlarged right jugulodigastric lymph node (2.9 × 2.5 cm). A CT scan of the chest showed mediastinal and bilateral hilar lymphadenopathy with bilateral interstitial nodular opacities in the upper lobes. A diagnostic PET scan showed the right jugulodigastric node to have a standardized uptake value (SUV) of 5.98 with bilateral oropharyngeal radiotracer activity. Surprisingly, radiotracer uptake in the oropharynx was higher in the left tonsil compared to the right. The patient underwent two rounds of panendoscopy with biopsies which revealed, respectively, mild dysplasia of the right tonsil and a friable and nodular inferior border of the right tonsil containing carcinoma in situ. Diagnostic right tonsillectomy showed extensive squamous cell carcinoma in situ with a high suspicion of invasion. The left tonsil was specifically noted to be clinically unremarkable.

**Figure 1:**
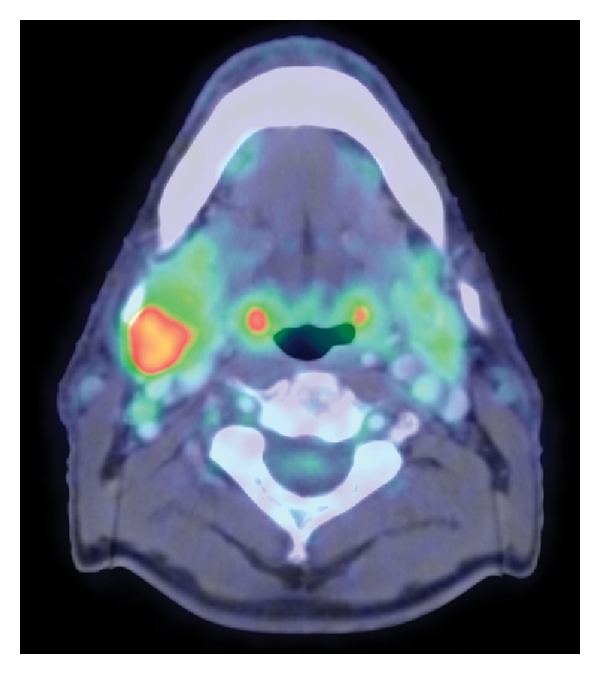
Axial view from fused PET/CT study demonstrates an enlarged right jugulodigastric node with heterogeneous but overall increased FDG avidity with peak SUV of 7.3.

**Figure 2:**
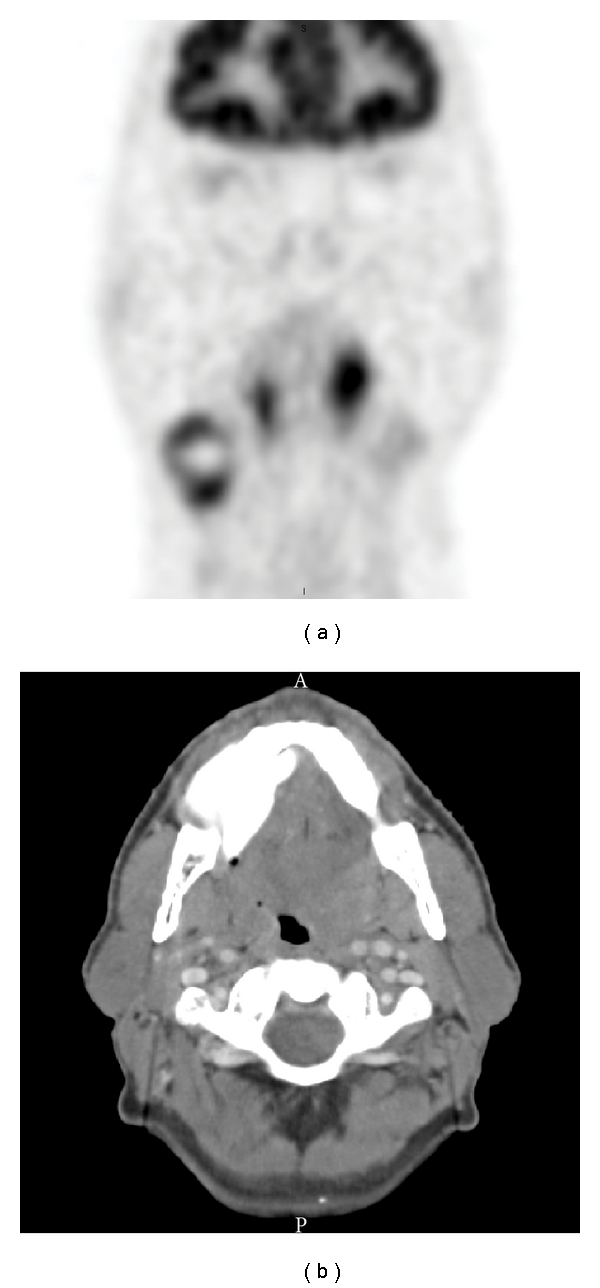
(a) Coronal FDG PET showing bilateral but asymmetric tonsillar FDG avidity. The patient had undergone diagnostic right tonsillectomy prior to this PET/CT study. However, peak SUV in the contralateral left palatine tonsil measured 8.7. (b) Axial CT showing pronounced left-sided tonsillar enlargement corresponding to the high FDG avidity.

**Figure 3:**
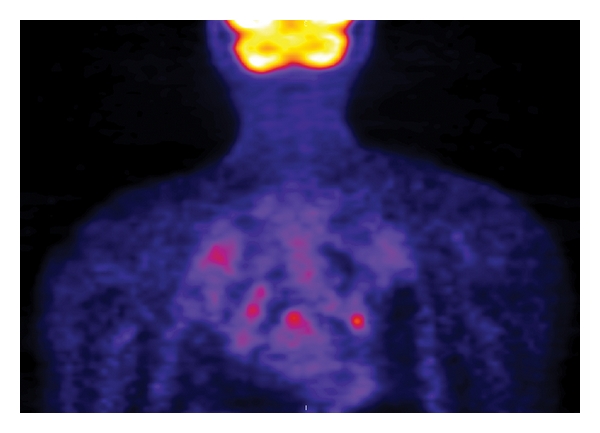
Coronal FDG-PET shows mild mediastinal and bilateral hilar FDG uptake, which is nonspecific but consistent with sarcoidosis.

**Figure 4:**
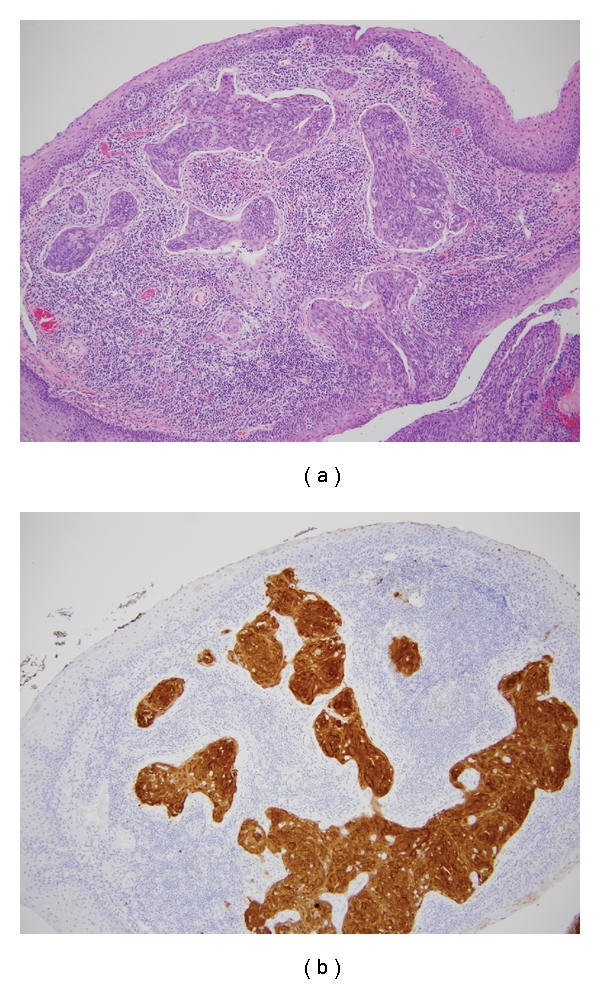
(a) Hematoxylin and eosin stained section of the left tonsil showing invasive squamous cell carcinoma, magnification 100X (b) Immunohistochemical stain for p16 performed on a serial section, magnification 100X.

A repeat PET/CT was performed for the purposes of radiotherapy planning and this study confirmed the presence of a 3 cm right neck level II lymph node with an SUV of 7.3, with central necrosis (Figure 1). A diagnostic head and neck contrast-enhanced CT was included as part of the PET/CT study, as is our institutional practice and review of the CT elicited concern for extracapsular spread. FDG-avidity was again noted bilaterally in the oropharynx, with the intact left tonsil showing a maximum SUV of 8.7 as compared to maximum SUV of 6.6 in the tonsillar bed on the right (Figure 2(a)). However, the left tonsil was noted to be unusually bulky and irregular on the CT portion of the imaging study (Figure 2(b)). The PET/CT also demonstrated mild, symmetric FDG avidity in the mediastinal and hilar regions, which was considered more consistent with an inflammatory rather than neoplastic process, given the low FDG uptake (Figure 3). High-resolution chest CT also suggested sarcoidosis. Due to the possibility that the asymmetric uptake and enlargement of the intact left tonsil might have been caused by the recent right tonsillectomy, the patient underwent a diagnostic left tonsillectomy. While the appearance of the left tonsil was still normal on examination, pathologic analysis revealed extensive in situ and invasive squamous cell carcinoma (Figure 4(a)). Immunohistochemical staining for p16 was diffusely and strongly positive (Figure 4(b)). Concurrent cisplatin was recommended due to the radiographically identified extracapsular extension, but the patient chose cetuximab in conjunction with intensity-modulated radiotherapy. Radiation treatment targeted the bilateral tonsillar beds and the right neck lymph node. The prescription dose was 6996 cGy, delivered over 33 fractions of treatment, with weekly localization using conebeam CT imaging to position the patient on the radiation therapy table. The concurrent cetuximab was delivered over 8 weekly infusion cycles and was well tolerated, with development of a moderate skin rash as is typical for this class of targeted therapy. After 2 years of followup, the patient is well with no evidence of recurrent cancer and his sarcoidosis remains stable.

## 3. Discussion

The exact diagnostic workup for CUPS remains a matter of debate. Examination under anesthesia is mandatory, but biopsy procedures are variable. Because a significant percentage of CUPS arise from the palatine tonsil [1–4], a typical recommendation is that an ipsilateral tonsillectomy be included in the standard workup [2, 3, 5–7]. However, a case series published by Koch et al. [8] found that the incidence of bilateral tonsillar cancers approaches 10%, leading to a recommendation for routine diagnostic bilateral tonsillectomy based on the goals of early control of all primary disease [5], avoidance of radiation to healthy tissues [3, 6], improved surveillance ability [6], and improved overall success rates [9, 10]. Yet despite probable benefit in a select number of patients, the practice of bilateral tonsillectomy remains controversial. 

The optimal imaging workup likewise remains undefined. A retrospective review found that PET/CT was superior to CT, MRI or whole body PET in primary site detection [9]. Compared to CT alone, PET/CT has higher sensitivity [4, 11], higher negative predictive value [4], and comparable [11] or inferior specificity [4] in the investigation of head and neck CUPS. Studies have estimated that the sensitivity of PET/CT may range from 66–87.5% with a specificity of 70–92.9% [4, 11–13]. A positive predictive value of 88.8% has been reported [12]. 

A retrospective review by Cianchetti et al. [7] included a proposed diagnostic algorithm for CUPS of the head and neck, including a complete physical exam, CT and/or MRI, panendoscopy with biopsies, PET/CT based on “indeterminate findings,” and unilateral or bilateral tonsillectomy in patients with extant lymphoid tissue [7]. In contrast, some authors have advocated for upfront PET/CT in order to direct the choice of biopsy sites at the time of panendoscopy, thereby increasing accuracy in detecting singular or synchronous primary cancers [14, 15]. We agree with the latter recommendation.

Of note, this patient was a lifelong nonsmoker and showed strong immunohistochemical staining for p16, consistent with association with high-risk human papillomavirus infection [16]. HPV-related oropharyngeal cancers treated with chemoradiation carry a superior prognosis, which is retained despite traditional indicators of aggressiveness such as regional nodal metastasis [17, 18]. One report did not find an increased propensity for bilateral tonsillar cancer in specimens that were positive for p16 immunohistochemistry and HPV-16 in situ hybridization [19]. However, other reports have speculated on the possibility of HPV-related field cancerization in Waldeyer's ring [20] or an HPV-related predisposition to the development of bilateral disease spread [21]. 

Finally, PET/CT findings may result in changes to staging or alteration of radiation therapy target volumes [13, 22–24]. A large prospective, blinded study specifically noted changes to the gross tumor volume, the extent of regional disease, the prescribed dose of radiation and/or the selection of treatment modality, leading to nearly a third of the patients' management being significantly altered [22]. One study found higher control rates and favorable toxicity profiles when utilizing PET/CT to guide radiotherapy [25]. 

Oncologists should be aware of the limitations of PET/CT in cases of head and neck CUPS. A high rate of false positive findings with PET/CT in the postoperative and preradiotherapy period has been shown [26]. Conditions such as infection, healing, or localized inflammatory processes as well as foreign bodies (catheters, prostheses) have been shown to produce false positive results on PET/CT [27]. Though rare, the so-called “sarcoid-like reaction to malignancy” should be considered when interpreting PET/CT [28]. F-18 fluorothymidine may have an advantage over F-18 fluorodeoxyglucose PET/CT scans for distinguishing sarcoidosis from malignancy [29]. 

Most importantly, the value of obtaining imaging studies of appropriate quality and technique should not be understated. While PET alone may be adequate for CUPS workup [30], other studies have found higher rates of sensitivity for PET/CT in identifying the primary site (up to 67%) as compared to historical rates reported from PET alone [31, 32], or with either PET or CT alone [33]. In the latter study, the imaging was interpreted by a team comprised of a nuclear medicine physician and a diagnostic radiologist [33]. Given the major changes in staging and therapy that result from identification of one or more head and neck primary sites, at our institution we recommend a dual-modality fused PET/CT including a diagnostic-quality head and neck contrast-enhanced CT, as part of the standard CUPS workup, ideally obtained prior to endoscopy and directed biopsies and/or bilateral tonsillectomy. We recommend evaluation by both a nuclear medicine and neuroradiology specialist to maximize the interpretative yield.

## 4. Conclusions

Skilled anatomic interpretation of PET/CT imaging led to the correct diagnosis of bilateral synchronous tonsillar squamous cell carcinoma. This case illustrates the expanding applications of PET/CT for head and neck cancer including the identification of occult or synchronous primaries in CUPS, detection of metastasis, and radiotherapy target identification. A properly executed PET/CT can greatly assist in the initial workup of patients with head and neck CUPS.

##  Conflict of Interests

The authors declare that there is no conflict of interests.
